# Supplementary material for: Identification and functional analysis of non-coding regulatory small RNA FenSr3 in Bacillus amyloliquefaciens LPB-18
Source: PeerJ. 2023 May 15;11:e15236. doi: 10.7717/peerj.15236 (PMC10194069; doi:10.7717/peerj.15236)
Supplement: Supplemental Information 4 [file peerj-11-15236-s004.zip › KO/CK-vs-T1_map/map04970.html]

KEGG PATHWAY: Salivary secretion - Reference pathway


|  |  |
| --- | --- |
| **Salivary secretion - Reference pathway** |  |

[
Pathway menu
| Organism menu
| Pathway entry
| Show description
| User data mapping
]

Saliva has manifold functions in maintaining the integrity of the oral tissues, in protecting teeth from caries, in the tasting and ingestion of food, in speech and in the tolerance of tenures, for example. Salivary secretion occurs in response to stimulation by neurotransmitters released from autonomic nerve endings. There are two secretory pathways: protein exocytosis and fluid secretion. Sympathetic stimulation leads to the activation of adenylate cyclase and accumulation of intracellular cAMP. The elevation of cAMP causes the secretion of proteins such as amylase and mucin. In contrast, parasympathetic stimulation activates phospholipase C and causes the elevation of intracellular Ca2+, which leads to fluid secretion; that is, water and ion transport. Ca2+ also induces amylase secretion, but the amount is smaller than that induced by cAMP.

|  |  |  |
| --- | --- | --- |
| Reference pathway | 184% 150% 122% 100% 82% 67% 55% | 图片下载 |
